# Enterovirus 71 Outbreaks, Taiwan: Occurrence and Recognition

**DOI:** 10.3201/eid0903.020285

**Published:** 2003-03

**Authors:** Tzou-Yien Lin, Shiing-Jer Twu, Mei-Shang Ho, Luan-Yin Chang, Chin-Yun Lee

**Affiliations:** * Chang Gung University, Taoyuan, Taiwan; † Chang Gung Children’s Hospital, Taoyuan, Taiwan; ‡ Ministry of Health, Taiwan; § Academia Sinica, Taipei, Taiwan; ¶ National Taiwan University Hospital, Taipei, Taiwan

**Keywords:** enterovirus 71, hand, foot, and mouth disease, outbreak, pulmonary edema, pathogenesis, neurovirulence, surveillance, vaccine, synopsis

Enterovirus 71 (EV71) caused a large outbreak in Taiwan in 1998 with 78 deaths, and smaller outbreaks recurred in 2000 and 2001. The outbreak was recognized because of a large number of hand, foot, and mouth disease cases and the rapid deaths of children with the disease. Virologic and pathologic studies indicated that EV71 was the most important agent related to severe and fatal cases and that a neurogenic inflammatory response was involved in the pathogenesis of cardiopulmonary collapse resulting from fulminant EV71 infection. Seroepidemiologic study suggested that EV71 had circulated for at least 16 years and that the accumulation of susceptible hosts might have triggered the 1998 outbreak. However, a change in EV71 neurovirulence and host genetic susceptibility may also have affected the clinical outcome. The Taiwan outbreak shows that worldwide attention should be paid to such outbreaks, new antiviral drugs should be developed, and that vaccination of children under 5 years of age may be warranted.

Enteroviruses consist of 68 serotypes and usually cause self-limited infections in children. Enterovirus 71 (EV71) was first isolated in California in 1969 (*1*). Since then, EV71 has been isolated in many parts of the world. Two patterns of EV71 outbreaks have been observed: small outbreaks associated with occasional patient death and severe outbreaks associated with a high case-fatality rate. The latter pattern occurred in Bulgaria in 1975 with 44 deaths (*2*) and Hungary in 1978 with 45 deaths (*3*). During the past 5 years severe outbreaks have occurred: in Malaysia in 1997 with 30 deaths and in Taiwan with 78 deaths in 1998, 25 deaths in 2000, and 26 deaths in 2001 (*4*).

## How Did the Taiwan Outbreaks Occur?

### Sentinel Surveillance Systems in Taiwan

In 1989, a physician-based sentinel surveillance system for infectious diseases was established in Taiwan and operated by the Ministry of Health. This system included 850 physicians, representing 8.7% of primary physicians in Taiwan. Hand, foot, and mouth disease (HFMD) and herpangina were included in the system after an outbreak of HFMD in Malaysia in 1997. When the case incidence after 1997 was compared with the case incidence of HFMD in 1997, the number of cases in Taiwan markedly increased after March 1998 (*5*). Because the numbers of severe and fatal cases of HFMD were increasing rapidly, a hospital-based reporting system for monitoring such cases was added in May 1998. Since June 1998, both physician-based and hospital-based surveillance systems have been maintained simultaneously (*5*).

## 1998 Outbreak

The 1998 outbreak occurred in two waves. The first and bigger wave peaked during the week of June 7 and encompassed all four regions of Taiwan. The second and smaller wave occurred during the week of October 4, mainly in southern Taiwan. The total number of HFMD and herpangina cases reported was 129,106 (*6*).

The severe cases of EV71 infection peaked in early June as well: 405 severe cases were reported with 78 deaths (*6*). Ninety one percent of patients with fatal cases were <5 years old (*6*). Most of these patients died of fulminant infection within 1 or 2 days of hospitalization. Of 96 patients with severe EV71 cases, 67 (70%) had encephalitis (*6*).

### Virologic and Pathologic Studies

Of 78 patients with fatal cases, 37 had a positive viral culture in which 34 (92%) yielded EV71 (*4*). In 1998, 177 strains (42%) of EV71, 73 strains (18%) of coxsackievirus A16, and 168 strains (40%) of other enteroviruses (*4*) were isolated in Chang Gung Children’s Hospital; only EV71 was isolated from patients who died or survived with chronic health problems (*4*). Thus, EV71 was the most important agent among fatal cases, although other enteroviruses also circulated during the outbreaks.

Pathologic studies showed extensive inflammation in the central nervous system (CNS), with predominant lesions in the brain stem and spinal cord (*7*). Marked pulmonary edema with focal hemorrhage occurred without evidence of myocarditis. EV71 was isolated from CNS tissues but not from other tissues. The autopsy findings were similar to those reported in Malaysia (*8*).

Clinical, virologic, and pathologic findings suggested that the CNS, especially the brain stem, is the primary area involved in severe EV71. We hypothesized that a neurogenic and systemic inflammatory response may be involved in the pathogenesis of cardiopulmonary collapse that occurs in severe EV71 infections (Figure). EV71 may invade the CNS through its presence in the blood or directly through the cranial nerves (facial or hypopharyngeal nerve) on day 2 to day 5 of illness. A severe inflammatory response occurs in the CNS, and we found a high interleukin 6 level (1326+/-389 pg/mL) in the cerebrospinal fluid on the first 2 days of CNS involvement (*9*), when cardiopulmonary collapse with pulmonary edema usually occurs. If appropriate cardiopulmonary supports are provided, patients may survive but usually have sequelae: for example, central hypoventilation; cranial nerve palsy such as dysphagia, abducens palsy, and facial palsy; and shoulder weakness and atrophy. These sequelae are compatible with magnetic resonance imaging findings, which usually reveal high signal intensity from the pons to the cervical spinal cord (*10*).

**Figure:**
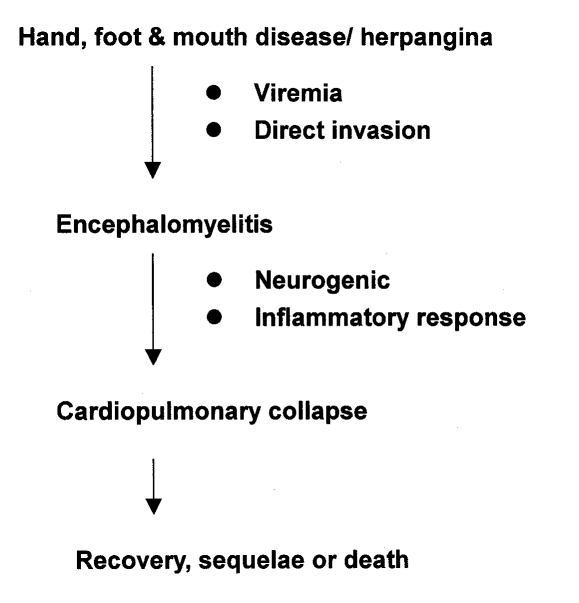
Pathogenesis of severe EV71 infections.

## Did EV71 Circulate in Taiwan Before 1998?

Chang et al. initiated a seropidemiologic study before and after the 1998 outbreak and found that in 1997 EV71 seroprevalence rates in adults and children >6 years of age were 57% to 67% (*11*). Lu et al. examined serial serum antibody titers to EV71 in 81 children born in 1988 who had yearly blood samples saved from 1989 to 1994 and in 1997 and 1999 (*12*). These researchers discovered that the incidence of EV71 seroconversion increased yearly from 3% to 11% between 1989 and 1997 and that 68% of these children had serologic evidence of EV71 infections by 1997 (*12*). In addition, EV71 had been isolated from patients with HFMD and poliomyelitis-like paralysis in Taiwan as early as 1981 and 1986 (*6*,*13*).

### How Was the 1998 Outbreak Recognized?

The evidence presented above indicated that EV71 had circulated in Taiwan for at least 16 years before 1998. However, its clinical significance in Taiwan was not investigated before 1998. Severe cases of EV71 probably had occurred but had not been recognized. In 1998, however, with the availability of specific EV71 monoclonal antibody, observant pediatricians in Taiwan linked the association of EV71 with young children dying of an unexplained acute illness. Both the physician-based and hospital-based surveillance systems supplied critical information leading to the recognition of the EV71 outbreaks and provided the opportunity for the study of EV71 infections.

## Why Did the 1998 Outbreak Occur?

The 1998 EV71 outbreak in Taiwan may have occurred for the following reasons:

l) mutation of the virus to a form with increased virulence; 2) presence of host factors—the accumulation of susceptible populations and individual genetic susceptibility. The outreak was recognized because of the establishment of a sentinel surveillance system (clinical and laboratory) and the awareness of health care workers.

### Outbreaks after 1998

One year after the large outbreaks in 1998, cases of EV71 infection decreased dramatically with only one fatal EV71 case in 1999. However, severe and fatal EV71 cases recurred, and 25 children died in 2000 and 26 in 2001 (Table). EV71 outbreaks likely will continue to occur for the next several years in Taiwan.

**Table:** Number of confirmed severe/fatal enterovirus infections and viral isolation results from patients with fatal cases, Taiwan, 1998–2000^a^

Case/enterovirus serotype	1998	1999	2000	2001
Severe cases	405	35	291	389
Fatal cases	78	9	41	55
Enterovirus 71	34	1	25	26
Coxsackievirus B3	0	3	1	0
Echovirus 4	0	0	0	3
Other enteroviruses	3	4	12	7
Negative	31	1	3	13
Specimens not available	10	0	0	6

^a^Data provided by the Center for Disease Control, Ministry of Health, Taiwan (1998–2001).

### Virus Studies

Wang et al. showed that most EV71 strains isolated in the 1998 outbreaks belonged to genotype C (*14*). In 1999 and 2000, however, genotype B was the most common strain. Recombination strains did not develop between the two genotypes, and particular EV71 genotypes did not affect clinical outcome (*14*).

Shih et al. analyzed the complete sequence of two selected EV71 strains (*15*), one from a fatal case and the other from an uncomplicated HFMD case. They found the identity to be similar (from 97% to 100%) both in amino acid and nucleotide sequence throughout the whole genome. Although the genetic identity of EV71 in the fatal case and the uncomplicated case was highly similar, their clinical outcomes were contrary.

Currently available genetic techniques cannot detect the EV71 genetic difference except the difference in genotype. As with polioviruses (*16*), a minor yet critical genetic change may have led to unusual neurovirulence and caused this outbreak.

### Host Factors

Chang et al. showed that preepidemic EV71 seroprevalence rates were inversely correlated with death and disease (*11*). Among a cohort of 81 children, Lu et al. found that the annual EV71 seroconversion rates (3% to 4%) between 1994 and 1997 were significantly lower than the rates (7% to 11%) before 1994 (*12*). The incidence of EV71 infection was likely lower between 1994 and 1997, and the accumulation of susceptible hosts over the threshold density of EV71 caused the 1998 outbreak.

Our preliminary data did not show a difference in tumor necrosis factor-α promoter polymorphism between uncomplicated and serious HFMD cases (unpub. data). We still believe that individual host genetic factors may affect clinical severity.

### Surveillance System

Pediatricians with a high index of suspicion and timely autopsy findings were the major factors that caused the outbreak to be recognized. Otherwise, this fulminant cardiopulmonary collapse would have been regarded as unexplained death. At present, no single factor can fully explain why a fairly common enterovirus can cause outbreaks of severe disease in selected countries of the world.

## Relationship between EV71 and HFMD and the Implications

In addition to the Bulgarian, Hungarian, Malaysian, and Taiwanese EV71 outbreaks, a study in Singapore also showed that the age-specific EV71 seroprevalence rate remained steady at approximately 50% among children >5 years old in 1996–1997 (*17*); EV71 also caused a polio-like syndrome after poliovirus was eradicated in Brazil (*18*), and the EV71 seropositive rate among 12- to 15-year-old Brazilian children was 69.2% (*19*). Given the lack of adequate viral diagnostic laboratories and the difficulty in isolating some strains of the virus, reports of EV71 infection may represent only the tip of the iceberg. Therefore, outbreaks of EV71 infection could spread to other parts of the world and cause substantial illness and death.

In conclusion, EV71 infection may have a fulminant clinical course, and particularly because approximately 90% of the fatal cases were in children <5 years of age, effective control measures must be implemented: new antiviral drugs should be developed and mass active vaccination of children <5 years old in disease-endemic areas should be considered.
